# Correction to: NoRCE: non‑coding RNA sets cis enrichment tool

**DOI:** 10.1186/s12859-021-04280-8

**Published:** 2021-08-04

**Authors:** Gulden Olgun, Afshan Nabi, Oznur Tastan

**Affiliations:** 1 Department of Computer Engineering, Bilkent University, Ankara, Turkey; 2 Present Address: Faculty of Engineering and Natural Sciences, Sabanci University, 34956 Istanbul, Turkey; 3 Present Address: Cancer Data Science Lab, National Cancer Institute, National Institute of Health, Bethesda, MD USA

## Correction to: BMC Bioinformatics (2021) 22:294 https://doi.org/10.1186/s12859-021-04112-9

Following the publication of the original article [1], the authors identified an error in the affiliation of Dr. Oznur Tastan. The correct affiliation is presented in the online version.

Correct affiliation:

Faculty of Engineering and Natural Sciences, Sabanci University, 34956, Istanbul, Turkey.

The original article [1] has been corrected.
