# Supplementary material for: NoRCE: non-coding RNA sets cis enrichment tool
Source: BMC Bioinformatics. 2021 Jun 2;22:294. doi: 10.1186/s12859-021-04112-9 (PMC8170991; doi:10.1186/s12859-021-04112-9)
Supplement: Supplementary file 1 — Additional file 1. Suplementary information and results for the package and further analysis. [file 12859_2021_4112_MOESM1_ESM.pdf]

Supplementary Notes for ‘NoRCE: Non-coding RNA Sets Cis Enrichment Tool’

Gulden Olgun[1], Afshan Nabi[2], Oznur Tastan[2*]
[1] Department of Computer Engineering, Bilkent University, Ankara, Turkey
[2] Faculty of Engineering and Natural Sciences, Sabanci University, Tuzla, Istanbul, Turkey
* otastan@sabanciuniv.edu

Contents

1 Supplementary Information on Methods **2**
1.1 Data Sources 2
1.1.1 Gene and Gene Ontology Annotations 2
1.1.2 TAD Boundaries Maintained by NoRCE 2
1.1.3 Details of the Custom Pathways 2
1.2 Data in NoRCE repository for the vignette and supplementary results 2

2 Supplementary Information on Results **4**
2.1 Case Study 1: Functional enrichment analysis of ncRNAs differentially expressed in psychiatric disorders 4
2.1.1 Functional enrichment results 4
2.1.2 Filtering the close-by genes according to TAD boundaries 6
2.1.3 TAD filtering enhancement 8
2.1.4 Pathway enrichment using predefined pathway gene sets 9
2.1.5 Comparison Between ASD Associated GO-terms and NoRCE Enrichment 13
2.2 Case Study 3: Functional enrichment analysis with co-expression analysis 14
2.3 Case Study 4: Functional enrichment analysis of pan-cancer driver lncRNAs filtered with TAD boundaries 15

1 Supplementary Information on Methods

1.1 Data Sources

NoRCE repository contains various datasets. Below, we detail these sources.

1.1.1 Gene and Gene Ontology Annotations

Table S1: The supported assemblies for different species in NoRCE.

Species	Supported Assembly	
	UCSC	NCBI
Homo sapiens	hg19	GRCh37, GCA_000001405.1, Feb. 2009
Homo sapiens	hg38	GRCh38, GCA_000001405.15, Dec. 2013
Mus musculus	mm10	GRCm38.p6, INSDC Assembly GCA_000001635.8, Jan 2012
Rattus norvegicus (brown rat)	rn6	Rnor_6.0, INSDC Assembly GCA_000001895.4, Jul 2014
Drosophila melanogaster (fruit fly)	dm6	BDGP6, INSDC Assembly GCA_000001215.4, Jul 2014
Danio rerio (zebrafish)	danRer10	GRCz10, GCA_000002035.3, Sep. 2014
Caenorhabditis elegans (worm)	ce11	WBcel235, GCA_000002985.3, Feb. 2013
Saccharomyces cerevisiae (yeast)	sacCer3	UCSC version sacCer3, 2011

1.1.2 TAD Boundaries Maintained by NoRCE

Table S2: Topological associating domain data that included in the NoRCE repository

Species	Name	Ver.	# of cell lines	# of TAD regions	Source
Homo sapiens	tad_hg19	hg19	37	74,424	3D Genome Browser[11]
Homo sapiens	tad_hg38	hg38	42	96,526	3D Genome Browser[11]
Mus musculus	tad_mm10	mm10	5	16,866	3D Genome Browser [11]
D. melanogaster	tad_dmel	dm6	1	2,846	HiCBrowser[7]

1.1.3 Details of the Custom Pathways

1.2 Data in NoRCE repository for the vignette and supplementary results

- brain_disorder_ncRNA : A list of ncRNAs differentially expressed in three psychiatric disorders including autism spectrum disorder (ASD), schizophrenia (SCZ), and bipolar disorder (BD) generated by Gandal et al. [3]. The set comprises1,363 ncRNAs. (Case study 1, Section 3.1).
- tad_custom : The TAD regions for dorsolateral prefrontal cortex obtained from [3]. The dataset contains 2,735 TAD regions. (Case study 1, Section 3.1).

Table S3: The number of pathways and the different pathway sources included in the January 2020 Bader Lab pathway data set.

Pathway Database	Number of Pathways
NetPath	25
IOB	33
Panther	173
NCI	223
Cyc	240
MSigdb	520
WikiPathways	559
Reactome	2,302
Total	4075

- miRCancerdb: The pre-computed Pearson correlation values of the expressions of pairs of $18,069,409$ miRNAs and genes for 34 cancer types. The data incorporates $1,015$ miRNAs, which are curated based on patient expression profiles that are provided by the TCGA. miRCancerdb is available at `https://figshare.com/articles/miRCancer_db_gz/5576329` [1]. (Case study 2, Section 3.2).

- brain_mirna : 407 differentially expressed miRNAs in human brain obtained from dbDEMC 2.0. (Case study 2, Section 3.2).

- breastmRNA : 667 differentially expressed mRNAs in human breast cancer. The analyze is conducted on patient expression data obtained from the TCGA project, collected on July 15th 2017. Differential expression analysis is applied for $p-value < 0.05$ and $FDR < 0.05$ using Bioconductor limma package[9].

- mirna & mrna : Subset of brain cancer expression levels for mRNA, and miRNA obtained from TCGA. Data contains 527 matched tumor patients for 150 mRNA and 183 miRNA. Those datasets are subset of the pre-processed miRNA and mRNA expression data that utilized in Case Study 2 and they are intented to use for examples.

- ncRegion : The relevant regions of the differentially expressed human ncRNA genes (except pseudogenes) for three psychiatric disorders. The diseases include autism spectrum disorder (ASD), schizophrenia (SCZ), and bipolar disorder (BD), and the data were generated by Gandal et al. [3]. The final set contains 930 gene regions.

2 Supplementary Information on Results

2.1 Case Study 1: Functional enrichment analysis of ncRNAs differentially expressed in psychiatric disorders

2.1.1 Functional enrichment results

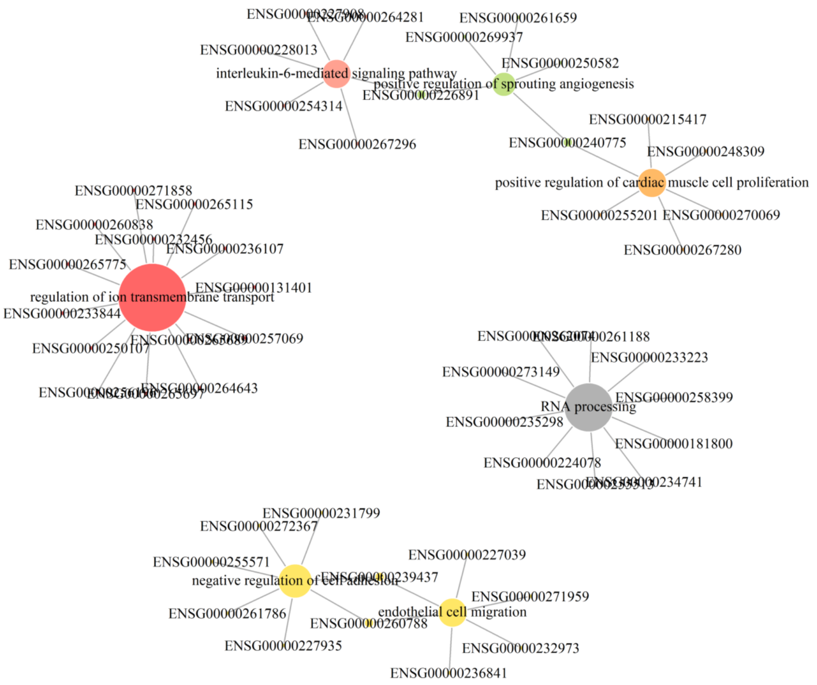

Figure S1: The top 7 enriched GO terms and the ncRNA genes which are associated with them are depicted as a network. The size of the nodes represents the degree, number of edges that are incident to the vertex, and each color represents different clusters in the network.

Table S4: Top 10 enrichment results of brain related biological process GO-terms that are obtained from the neighbourhood coding genes of ncRNA set of the [3]. The GeneRatio is computed by dividing the overlapping with the coding genes with the functional gene set to the number of all protein-coding genes within the input set neighbourhood. The BGRatiao column represents the ratio of the number of genes found in the enriched GO term set to the size of the background gene set. The EGNo refers to the size of the overlap between the corresponding GO term gene set and the neighboring coding gene set. ncGeneList column contains ncRNA genes that are enriched with the corresponding GO-term.

ID	Term	Pvalue	GeneRatio	BGRatio	EGNo	ncGeneList
GO:0006396	RNA processing	1.984e-30	86/692	552/18671	86	CELF2-AS1 AL354733.3 AL117190.1 SNHG14 GAS5 SNORD3B-2 AC016876.1 AC005363.1 Z95115.1 RP11-290D2.6
GO:0070102	interleukin-6-mediated signaling pathway	2.142e-4	5/692	16/18671	5	IL6R-AS1 AC021915.2 AC016596.2 LINC01359 CTD-2031P19.3 CEBPA-AS1
GO:0043542	endothelial cell migration	5.806e-4	6/692	29/18671	6	ITGB2-AS1 CYP1B1-AS1 AC007750.1 RN7SL752P AC009063.2 AC100803.4
GO:1903672	positive regulation of sprouting angiogenesis	1.976e-3	5/692	25/18671	5	AC021205.1 SMAD1-AS2 AC093525.7 LINC01359 Z92544.1
GO:0007162	negative regulation of cell adhesion	2.065e-3	7/692	49/18671	7	AL390243.1 PA2G4P6 RN7SL752P MIR9-3HG AC009063.2 CTC-428H11.2 AC006058.1
GO:0060045	positive regulation of cardiac muscle cell proliferation	2.544e-3	6/692	38/18671	6	MIR17HG AC021205.1 MEF2C-AS1 AC087623.1 TBX2-AS1 MIR222HG
GO:0034765	regulation of ion transmembrane transport	3.165e-3	11/692	113/18671	11	KCNQ5-IT1 SCN1A-AS1 CACNA1G-AS1 AP003721.1 KCNK4-TEX40 AC024614.1 AC011824.1 AC024614.2 AC011824.3 AC004147.5 Z84492.1 NAPSB AL355994.2 AC022893.2
GO:0035690	cellular response to drug	4.928e-3	7/692	57/18671	7	PPM1F-AS1 AP000320.1 MEF2C-AS1 RP11-199F11.2 AC005197.1 MT1L AL158206.1
GO:1902895	positive regulation of pri-miRNA transcription by RNA polymerase II	5.262e-3	5/692	31/18671	5	SREBF2-AS1 TGFB2-AS1 SMAD1-AS2 RP11-199F11.2 RN7SL192P AC011442.1
GO:0001525	angiogenesis	5.989e-3	17/692	232/18671	17	AL135960.1 RPL23AP74 EPHA1-AS1 TGFB2-AS1 CYP1B1-AS1 AC007750.1 UNC5B-AS1 RN7SL752P PTP4A2P2 AC087623.1 AC025569.1 AC020909.3 AC007193.2 RAB11B-AS1 KIF9-AS1 TBX2-AS1 PACERR RP11-383I23.2

2.1.2 Filtering the close-by genes according to TAD boundaries

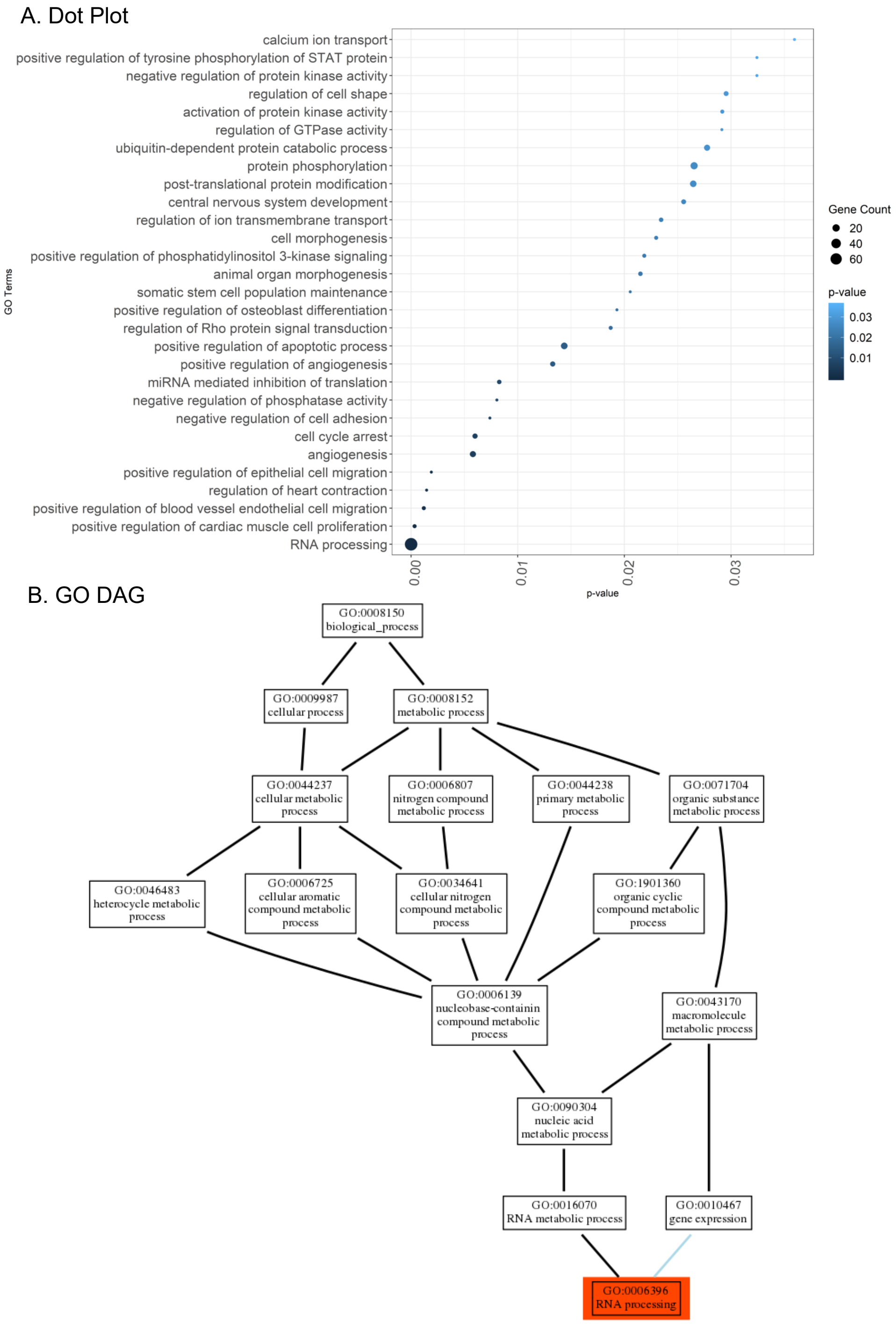

Figure S2: GO enrichment results for the genes that are in the same TAD boundaries with their close-by non-coding RNAs are only used. A) Top 35 GO biological process enrichment results pertaining to differentially expressed brain ncRNAs. The x-axis represent the p-value; the y-axis shows the GO term. The dot area is proportional to the size of the overlapping gene set, and the color signifies the p-value of the enrichment test for the corresponding GO-term. B) GO DAG diagram for the most enriched GO term. Enriched GO- terms are colored according to the p-value.

Table S5: Top 10 biological process GO term enrichment results when TAD filtering is used. The TAD information is from adult dorsolateral prefrontal cortex data [3] study. The GeneRatio is computed by dividing the overlapping with the coding genes with the functional gene set to the number of all protein-coding genes within the input set neighbourhood. The BGRatio column represents the ratio of the number of genes found in the enriched GO term set to the size of the background gene set. The EGNo refers to the size of the overlap between the corresponding GO term gene set and the neighboring coding gene set. ncGeneList column contains ncRNA genes that are enriched with the corresponding GO-term.

ID	Term	Pvalue	GeneRatio	BGRatio	EGNo	ncGeneList
GO:0006396	RNA processing	1.417e-36	78/467	552/18671	78	CELF2-AS1 AL354733.3 AL117190.1 SNHG14 GAS5
GO:0060045	positive regulation of cardiac muscle cell proliferation	3.179e-4	6/467	38/18671	6	MIR17HG AC021205.1 MEF2C-AS1 AC087623.1 TBX2-AS1 MIR222HG
GO:0043536	positive regulation of blood vessel endothelial cell migration	1.195e-3	6/467	48/18671	6	AC079305.1 AC021205.1 MIR210HG AC087623.1 AC093525.7 MIR222HG
GO:0008016	regulation of heart contraction	1.465e-3	5/467	34/18671	5	CELF2-AS1 AC020907.2 AC003991.1 AC074212.1 TBX2-AS1 DM1-AS
GO:0010634	positive regulation of epithelial cell migration	1.905e-3	5/467	36/18671	5	PPM1F-AS1 TGFB2-AS1 SOX9-AS1 MIR222HG
GO:0001525	angiogenesis	5.795e-3	13/467	232/18671	13	AL135960.1 RPL23AP74 EPHA1-AS1 TGFB2-AS1 CYP1B1-AS1 AC007750.1 UNC5B-AS1 RN7SL752P PTP4A2P2 AC087623.1 AC025569.1 AC020909.3 AC007193.2 TBX2-AS1
GO:0007050	cell cycle arrest	5.994e-3	9/467	132/18671	9	TGFB2-AS1 PRKAG2-AS1 SOX2-OT IL12A-AS1 AC002070.1 AC008691.1 AC127496.1 AC002546.1 tRP1-193H18.2 AC116667.1
GO:0007162	negative regulation of cell adhesion	7.394e-3	5/467	49/18671	5	AL390243.1 PA2G4P6 RN7SL752P MIR9-3HG AC009063.2
GO:0010923	negative regulation of phosphatase activity	8.050e-3	5/467	50/18671	5	MIR22HG PPP1R26-AS1 ELFN2 AC130404.1 AC079336.2 AC079336.3
GO:0035278	miRNA mediated inhibition of translation	8.268e-3	7/467	92/18671	7	MIR181A1HG MIR210HG MIR100HG MIR9-3HG MIR222HG

2.1.3 TAD filtering enhancement

Table S6: Brain disorder related biological process GO term enrichment results that show the TAD analysis enhancement. The listed enriched GO terms that are detected by enrichment analysis based on neighboring genes and TAD filtering can not be detected by enrichment analysis based on only neighboring genes. These findings prove the improvement when different analyses are used. The TAD information is obtained from adult dorsolateral prefrontal cortex data [3] study.

ID	Term	Pvalue	Reference
GO:0035023	regulation of Rho protein signal transduction	0.0187	[5, 4]
GO:0035019	somatic stem cell population maintenance	0.0205	[5]
GO:0006816	calcium ion transport	0.0360	[5]
GO:0014068	positive regulation of phosphatidylinositol 3-kinase signaling	0.0219	[10, 2, 5]
GO:0006511	ubiquitin-dependent protein catabolic process	0.0277	[8, 5]
GO:0007417	central nervous system development	0.0255	[12, 6]

2.1.4 Pathway enrichment using predefined pathway gene sets

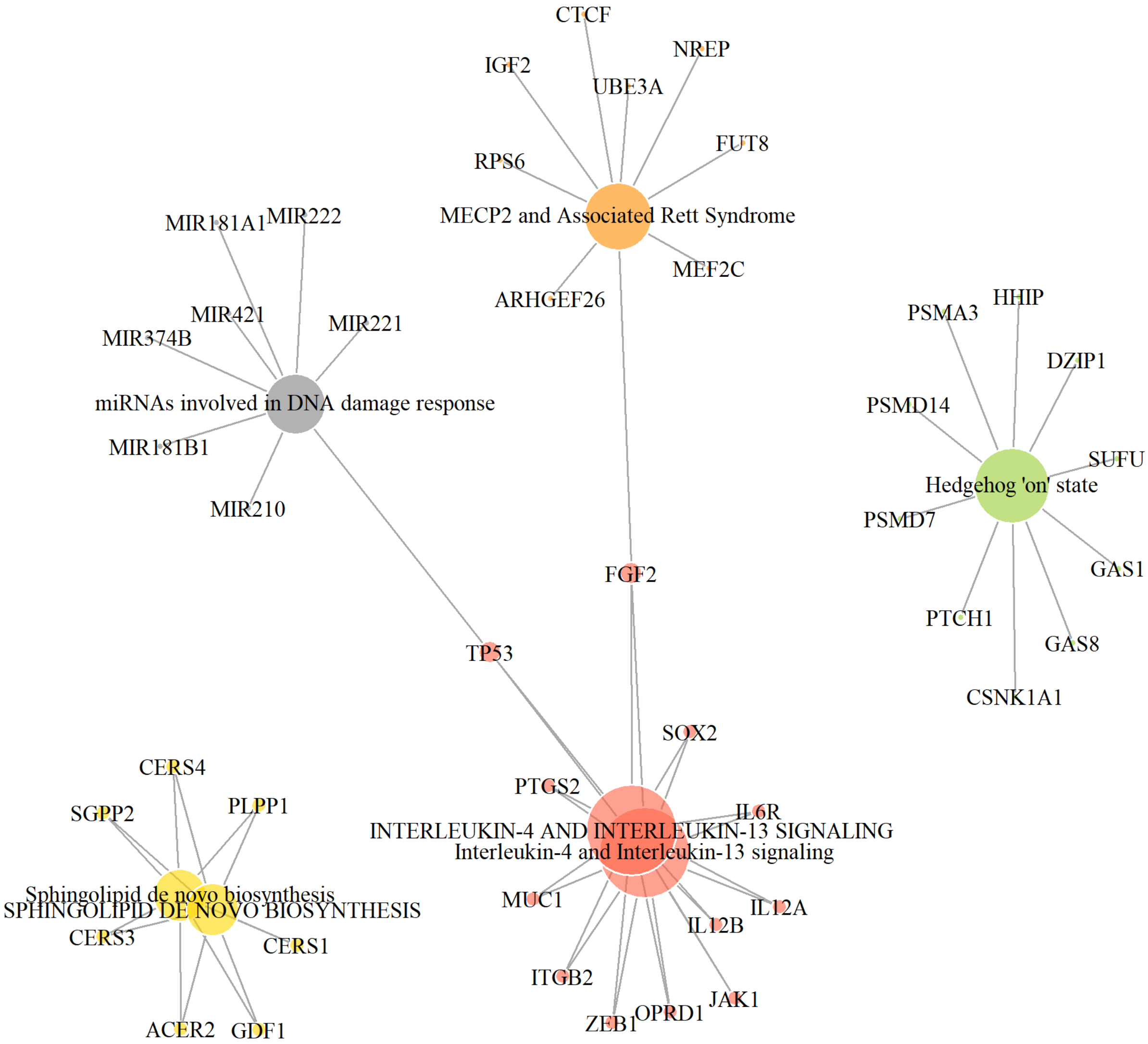

Figure S3: Interaction between the top 7 enriched pathways and the genes are depicted as a network. The size of the nodes represents the degree, number of edges that are incident to the vertex, and each color represents different clusters in the network.

Table S7: Neural disease related pathway enrichment results for the brain disease related ncRNA genes using customized pathway databases. If same pathway is detected in multiple database, we list the enriched pathway that have smaller p-adjusted value. The GeneRatio is computed by dividing the overlapping with the coding genes with the functional gene set to the number of all protein-coding genes within the input set neighbourhood. The BGRatio column represents the ratio of the number of genes found in the enriched GO term set to the size of the background gene set. The EGNo refers to the size of the overlap between the corresponding GO term gene set and the neighboring coding gene set. ncGeneList column contains ncRNA genes that are enriched with the corresponding GO-term.

Pathway Term	Database	Pathway ID	Pvalue	GeneRatio	BGRatio	EGNo	ncGeneList
miRNAs involved in DNA damage response	WikiPathways	WP1545	3.489e-4	8/484	50/13604	8	MIR181A1HG FTX MIR210HG RP11-199F11.2 MIR222HG RP3-368A4.5 RP3-368A4.6
INTERLEUKIN-4 AND INTERLEUKIN-13 SIGNALING	REACTOME	R-HSA-6785807	5.793e-4	12/484	111/13604	12	ITGB2-AS1 IL6R-AS1 ZEB1-AS1 AC021205.1 SOX2-OT IL12A-AS1 AC008691.1 RP11-199F11.2 RP1-212P9.2 LINC01359 THBS3-AS1 PACERR
SPHINGOLIPID DE NOVO BIOSYNTHESIS	REACTOME	R-HSA-1660661	7.325e-4	7/484	43/13604	7	AC005197.1 AC022146.2 AL158206.1 AC027020.2
MECP2 and Associated Rett Syndrome	WikiPathways	WP3584	8.890e-4	9/484	71/13604	9	RPL21P8 AC021205.1 AC132217.1 ARHGEF26-AS1 STARD4-AS1 MEF2C-AS1 SNHG14 AC027682.4 AL391834.2
Alzheimers Disease	WikiPathways	WP2059	9.268e-4	14/484	150/13604	14	MIR181A1HG LINC00461 AC087854.1 MIR124-1HG MIR100HG MIR9-3HG RP11-199F11.2 CASP4LP AC008525.1 KCNK4-TEX40 AL117190.1 AC006058.1 CTC-344H19.4
HEDGEHOG 'ON' STATE	REACTOME	R-HSA-56326846	0.0009	10/484	86/13604	10	URAHP MXRA7P1 HHIP-AS1 PSMA3-AS1 AC021078.1 AL161729.2 GAS1RR AC009120.1 DNAJC3-DT AL391121.1
SIGNALING BY HEDGEHOG	REACTOME	R-HSA-5358351	0.0012	13/484	137/13604	13	URAHP MXRA7P1 HHIP-AS1 PSMA3-AS1 AC021078.1 AC007780.1 AL161729.2 GAS1RR SCN1A-AS1 AC009120.1 DNAJC3-DT SEPTIN7P2 AL391121.1

Table S8: Continued Table-Neural disease related pathway enrichment results for the brain disease related ncRNA genes using customized pathway databases. The GeneRatio is computed by dividing the overlapping with the coding genes with the functional gene set to the number of all protein-coding genes within the input set neighbourhood. The BGRatio column represents the ratio of the number of genes found in the enriched GO term set to the size of the background gene set. The EGNo refers to the size of the overlap between the corresponding GO term gene set and the neighboring coding gene set. ncGeneList column contains ncRNA genes that are enriched with the corresponding GO-term.

Pathway Term	Database	Pathway ID	Pvalue	GeneRatio	BGRatio	EGNo	ncGeneList
Sphingolipid Metabolism (general overview)	WikiPathways	WP4725	0.0013	5/484	24/13604	5	AC005197.1 AC022146.2 RP11-16P6.1 AC027020.2
Sphingolipid Metabolism (integrated pathway)	WikiPathways	WP4726	0.0016	5/484	25/13604	5	AC005197.1 AC022146.2 RP11-16P6.1 AC027020.2
Prion disease pathway	WikiPathways	WP3995	0.0018	6/484	37/13604	6	MEF2C-AS1 AC087623.1 AC027682.4 CASP4LP LINC02202 AP000880.1
Amyotrophic lateral sclerosis (ALS)	WikiPathways	WP2447	0.0021	6/484	38/13604	6	AC087854.1 RP11-199F11.2 AC002546.1 AF106564.1 CASP4LP KCNK4-TEX40 RP1-193H18.2
Prader-Willi and Angelman Syndrome	WikiPathways	WP3998	0.0023	8/484	66/13604	8	CCND2-AS1 RP11-566K19.6 SNHG14 RP11-199F11.2
AXON GUIDANCE	REACTOME	R-HSA-422475	0.0023	32/484	528/13604	32	RP11-651P23.2 AC004057.1 GNRHR2 AL390243.1 EPHA1-AS1 AL133384.1 AC139149.1 PA2G4P6 MXRA7P1 SCN1A-AS1 MFF-DT UNC5B-AS1 RN7SL752P AC117395.1 CACNA1G-AS1 AC097381.2 AC113133.1 AC005363.1 PSMA3-AS1 AC022336.2 AC009065.4 CTC-428H11.2 AC079834.1 AL391834.2 RP5-886K2.1 AL390719.1 PRKCQ-AS1 AC009120.1 DRAIC AP000880.1 RP5-1024G6.8 RP11-54O7.18

Table S9: Continued Table-Neural disease related pathway enrichment results for the brain disease related ncRNA genes using customized pathway databases. The GeneRatio is computed by dividing the overlapping with the coding genes with the functional gene set to the number of all protein-coding genes within the input set neighbourhood. The BGRatio column represents the ratio of the number of genes found in the enriched GO term set to the size of the background gene set. The EGNo refers to the size of the overlap between the corresponding GO term gene set and the neighboring coding gene set. ncGeneList column contains ncRNA genes that are enriched with the corresponding GO-term.

Pathway Term	Database	Pathway ID	Pvalue	GeneRatio	BGRatio	EGNo	ncGeneList
Phosphodiesterases in neuronal function	WikiPathways	WP4222	0.0029	7/484	54/13604	7	PART1 AC109486.1 SETP21 PDE9A-AS1 SNX2P2 PDE6B-AS1 AC093752.1 RP11-382B18.4 SEPTIN7P2
ACTIVATION OF NMDA RECEPTORS AND POSTSYNAPTIC EVENTS	REACTOME	R-HSA-442755	0.0033	8/484	70/13604	8	RP11-204C16.4 PPM1F-AS1 PRKAG2-AS1 CKMT2-AS1 AC002070.1 AC007780.1 AC093525.7 SEPTIN7P2
Synaptic signaling pathways associated with autism spectrum disorder	WikiPathways	WP4539	0.0091	6/484	51/13604	66	PRKAG2-AS1 AC002070.1 SNHG14 AC127496.1 AP006545.1 AP006547.1
Signaling Pathways in Glioblastoma	WikiPathways	WP2261	0.0092	8/484	83/13604	8	RPL36AP15 AC087623.1 CCND2-AS1 AL355916.2 RP11-199F11.2 AC002546.1 AC093525.7 PRKCQ-AS1 RP1-193H18.2

2.1.5 Comparison Between ASD Associated GO-terms and NoRCE Enrichment

Table S10: List of ASD-associated GO-terms that are identified by the NoRCE is provided for each analysis. ORatio column represents the observed ratio which is a fraction of the number of observed GO-terms to total number of identified GO-terms.

Analysis	ORatio	GO-terms
Close-by Genes	32/48	GO:0070102 GO:0043542 GO:0007162 GO:0043542 GO:0007162 GO:0060045 GO:0034765 GO:0001525 GO:0008016 GO:0043536 GO:0045766 GO:0007050 GO:0035335 GO:0030148 GO:0006890 GO:0000209 GO:0051216 GO:0035278 GO:0032147 GO:0045669 GO:0016339 GO:0071407 GO:0008344 GO:0010923 GO:0045668 GO:0043087 GO:0008360 GO:0006470 GO:0006417 GO:0019233 GO:0006469 GO:0006887
TAD based	21/29	GO:0060045 GO:0043536 GO:0008016 GO:0010634 GO:0001525 GO:0007050 GO:0007162 GO:0010923 GO:0035278 GO:0045766 GO:0035023 GO:0045669 GO:0035019 GO:0014068 GO:0034765 GO:0006511 GO:0043087 GO:0032147 GO:0008360 GO:0006469 GO:0006816

2.2 Case Study 3: Functional enrichment analysis with co-expression analysis

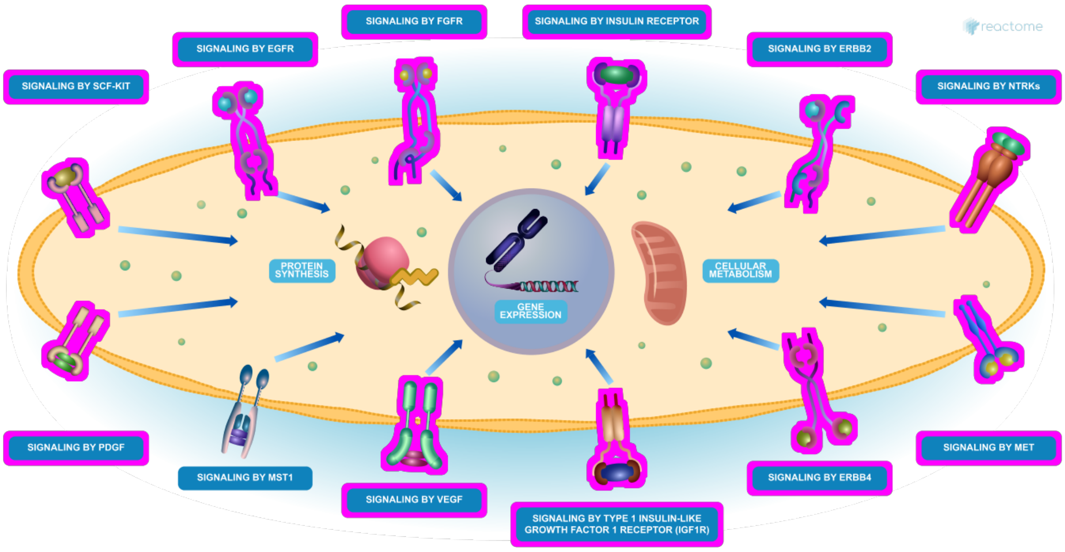

Figure S4: Reactome Diagram for the identified enriched pathway, Signaling by Receptor Tyrosine Kinases. For the given miRNA, coding genes that pass all filters for this analysis are flagged with purple color. Also, if those genes are annotated with another pathway in the diagram, positive pathways are also marked with purple color.

2.3 Case Study 4: Functional enrichment analysis of pan-cancer driver lncRNAs filtered with TAD boundaries

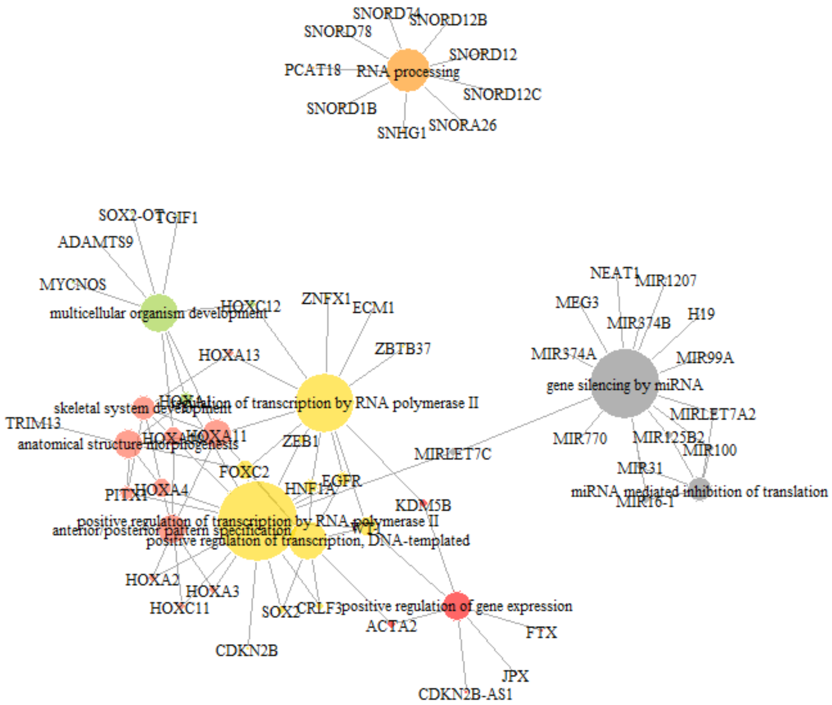

Figure S5: Network for all enriched processes for CLC. The nodes' size represents the degree, the number of edges that are incident to the vertex, and each color represents different clusters in the network.

Table S11: All biological process GO term enrichment results when TAD filtering is used in CLC data. The TAD information is obtained from 3D Genome Browser[11]. The GeneRatio is computed by dividing the overlapping with the coding genes with the functional gene set to the number of all protein-coding genes within the input set neighbourhood. The BGRatio column represents the ratio of the number of genes found in the enriched GO term set to the size of the background gene set. The EGNo refers to the size of the overlap between the corresponding GO term gene set and the neighboring coding gene set. ncGeneList column contains ncRNA genes that are enriched with the corresponding GO-term.

ID	Term	Pvalue	GeneRatio	BGRatio	EGNo	ncGeneList
GO:0035195	gene silencing by miRNA	5.47E-06	14/107	578/18671	14	H19 MIR31HG MEG3 LINC00478 FTX DLEU2 NEAT1 PVT1 RP11-820L6.1
GO:0009952	anterior/posterior pattern specification	1.99E-05	6/107	97/18671	6	HOTAIR HOTAIRM1 HOXA11-AS HOXA-AS2
GO:0009653	anatomical structure morphogenesis	3.67E-05	6/107	108/18671	6	HOTAIRM1 HOXA11-AS HOXA-AS2 ODRUL DLEU2 Epist
GO:0035278	miRNA mediated inhibition of translation	0.00018626	5/107	92/18671	5	MIR31HG LINC00478 DLEU2 RP11-820L6.1
GO:0045944	positive regulation of transcription by RNA polymerase II	0.00071757	16/107	1134/18671	16	WT1-AS LINC00478 EGFR-AS1 HOTAIR HOTAIRM1 ZEB1-AS1 ANRIL HNF1A-AS1 SOX2OT HOXA-AS2 ODRUL SUZ12P1 HOXA11-AS Epist
GO:0001501	skeletal system development	0.001081274	5/107	135/18671	5	HOXA11-AS HOTTIP HOXA-AS2 Epist
GO:0007275	multicellular organism development	0.00376224	8/107	439/18671	8	ENSG00000223850 HOTAIRM1 HOXA11-AS ADAMTS9-AS2 SOX2OT HOTAIR GAPLINC
GO:0006396	RNA processing	0.004457922	9/107	552/18671	9	NcRAN ZFAS1 DANCR GAS5 SNHG1
GO:0045893	positive regulation of transcription, DNA-templated	0.015968489	8/107	565/18671	8	ZXF1 WT1-AS EGFR-AS1 HOXA11-AS HNF1A-AS1 SOX2OT ODRUL SUZ12P1
GO:0010628	positive regulation of gene expression	0.023457714	6/107	385/18671	6	ZXF1 WT1-AS FTX ANRIL PCAN-R1
GO:0006357	regulation of transcription by RNA polymerase II	0.031750673	12/107	1150/18671	12	ZFAS1 WT1-AS EGFR-AS1 GAS5 ZEB1-AS1 HOXA11-AS HNF1A-AS1 HOTTIP ODRUL FAL1 PCAN-R1 HOTAIR

References

[1] M. Ahmed, H. Nguyen, T. Lai, and D. R. Kim. mircancerdb: a database for correlation analysis between microrna and gene expression in cancer. *BMC research notes*, 11(1):103, 2018.

[2] D. A. Fruman, H. Chiu, B. D. Hopkins, S. Bagrodia, L. C. Cantley, and R. T. Abraham. The pi3k pathway in human disease. *Cell*, 170(4):605–635, 2017.

[3] M. J. Gandal, P. Zhang, E. Hadjimichael, R. L. Walker, C. Chen, S. Liu, H. Won, H. van Bakel, M. Varghese, Y. Wang, et al. Transcriptome-wide isoform-level dysregulation in asd, schizophrenia, and bipolar disorder. *Science*, 362(6420):eaat8127, 2018.

[4] N. N. Kasri and L. Van Aelst. Rho-linked genes and neurological disorders. *Pflügers Archiv-European Journal of Physiology*, 455(5):787–797, 2008.

[5] e. a. Krishnan. Arjun. Genome-wide prediction and functional characterization of the genetic basis of autism spectrum disorder. *Nature neuroscience*, 19(11):1454, 2016.

[6] A. Kumar, C. C. Swanwick, N. Johnson, I. Menashe, S. N. Basu, M. E. Bales, and S. Banerjee-Basu. A brain region-specific predictive gene map for autism derived by profiling a reference gene set. *PloS one*, 6(12):e28431, 2011.

[7] F. Ramírez, V. Bhardwaj, L. Arrigoni, K. C. Lam, B. A. Grüning, J. Villaveces, B. Habermann, A. Akhtar, and T. Manke. High-resolution tads reveal dna sequences underlying genome organization in flies. *Nature communications*, 9(1):189, 2018.

[8] G. Rao, B. Croft, C. Teng, and V. Awasthi. Ubiquitin-proteasome system in neurodegenerative disorders. *Journal of drug metabolism & toxicology*, 6(4), 2015.

[9] M. E. Ritchie, B. Phipson, D. Wu, Y. Hu, C. W. Law, W. Shi, and G. K. Smyth. limma powers differential expression analyses for rna-sequencing and microarray studies. *Nucleic acids research*, 43(7):e47–e47, 2015.

[10] K. Sánchez-Alegría, M. Flores-León, E. Avila-Muñoz, N. Rodríguez-Corona, and C. Arias. Pi3k signaling in neurons: a central node for the control of multiple functions. *International journal of molecular sciences*, 19(12):3725, 2018.

[11] Y. Wang, F. Song, B. Zhang, L. Zhang, J. Xu, D. Kuang, D. Li, M. N. Choudhary, Y. Li, M. Hu, et al. The 3d genome browser: a web-based browser for visualizing 3d genome organization and long-range chromatin interactions. *Genome biology*, 19(1):151, 2018.

[12] M. N. Ziats and O. M. Rennert. Expression profiling of autism candidate genes during human brain development implicates central immune signaling pathways. *PloS one*, 6(9):e24691, 2011.
